# Novel Relationship Between Hemoglobin A1c Levels and Foot Ulcer Development Among Patients With Type 2 Diabetes Mellitus Admitted at Tupua Tamasese Meaole Hospital

**DOI:** 10.7759/cureus.20054

**Published:** 2021-11-30

**Authors:** Rustum M Moodley, Stacey S D'Almeida, Viali Lameko, Suraj Ghimire

**Affiliations:** 1 Medicine, Oceania University of Medicine, Apia, WSM; 2 Internal Medicine, Oceania University of Medicine, Apia, WSM

**Keywords:** samoa, internal medicine, hemoglobin a1c, foot ulcers, diabetes

## Abstract

Background: Diabetes mellitus is one of the leading chronic conditions worldwide. One of its most debilitating complications is diabetic foot ulcers (DFUs), which appear to have an increased incidence in the Pacific Islands. However, this report has not been studied extensively in Samoa. Nevertheless, DFUs may be prevented through strict glycemic control by hemoglobin A1c (HbA1c) level monitoring.

Objective: This study aimed to identify a specific cutoff point for HbA1C to reduce the occurrence of DFUs in patients with type 2 diabetes mellitus (T2DM) admitted to an internal medicine ward in Samoa. Increased HbA1c levels are hypothesized to be strongly associated with DFU development.

Methods: A retrospective unmatched case-control study examined 100 patients with T2DM (50 patients with DFUs [case] and 50 patients without DFUs [control]) over four months. Participants were selected by convenience sampling.

Results: The HbA1c results were available in 32 cases and 29 controls. The receiver operating characteristic curve showed that the area under the curve was 51% (95% CI, 36%-66%; standard error, 0.075; *P* = 0.8966), and no cutoff point could be established.

Conclusion: The HbA1c is not an ideal test to readily predict DFUs in patients with T2DM.

## Introduction

Diabetes mellitus (DM) is a chronic condition of impaired blood glucose control that can ultimately lead to microvascular and macrovascular complications. In DM, the body cannot produce enough insulin, or the cells are unresponsive to the insulin produced. DM is diagnosed by a random blood glucose (RBG) level greater than 200 mg/dL (11.1 mmol/L) or a fasting blood glucose level greater than 126 mg/dL (7 mmol/L). It is also diagnosed by a 2-hour oral glucose tolerance test level of 200 mg/dL (11.1 mmol/L) or a hemoglobin Ab1c (HbA1c) level greater than 6.5 mmol/L [1]. The HbA1c level is a measure of glycemic control for three months and is currently the recommended diagnostic test for type 2 diabetes (T2DM) [2]. However, other glycemic control biomarkers can be considered instead of the HbA1c test. These glycemic control biomarkers are particularly important in conditions such as chronic kidney disease. In chronic kidney disease, the overall production of red blood cells is decreased because of erythropoietin reduction; therefore, HbA1c cannot be used reliably. Glycemic biomarkers such as glycated albumin and fructosamine indicate the mean blood glucose concentration during the lifespan of total plasma albumin or proteins; their lifespan is roughly three weeks [3]. Thus, these tests can be utilized in hemoglobinopathies and anemia, where HbA1c has decreased efficacy [4]. One of the most devastating complications of DM is diabetic foot ulcers (DFUs). DFU refers to a full-thickness wound through the dermis and found right below the ankle on a weight-bearing surface in an individual with DM [5]. Its pathogenesis involves repetitive injury to a poorly vascularized or insensate foot [6]. Calluses, underlying peripheral neuropathy, impaired circulation, and poor glycemic control are its most common risk factors.

Although DM treatment has remarkably improved in recent years, DFUs continue to be a crucial international burden for patients and the healthcare systems, specifically in resource-limited settings [7]. Approximately a quarter of the total healthcare expenditure in the diabetes population is related to foot complications [8]. In Australia, 1.9%-5.3% of people with DM have experienced DFUs [9], which disproportionately affects the indigenous and socially disadvantaged populations in this country. The longer an individual has had poorly controlled blood glucose levels, the higher the risk for DFUs and amputation. The lifetime risk of developing DFUs is approximately 25%, and the risk of re-ulceration is roughly 65% within 5 years [10].

According to a recent study, foot ulcerations are almost entirely preventable through simple interventions such as monitoring long-term glycemic control [6]. Therefore, the HbA1c has recently surfaced as one of the prospective modifiable risk factors that are repeatedly elevated in patients with DFUs [11]. However, its evidence in the literature is very limited. High HbA1c levels are associated with increased microvascular complications, such as retinopathy, neuropathy, and nephropathy. One study attempted to identify the threshold level of HbA1c to predict certain microvascular complications, including mild and moderate retinopathy, which obtained an optimal cutoff point between 6.6% and 7%; unfortunately, these cutoff points could not detect any retinopathy, albuminuria, chronic kidney disease, and peripheral neuropathy [12]. Microvascular complications, such as neuropathy, predispose patients with diabetes to DFUs. HbA1c and neuropathy are independent predictors of foot ulceration [13].

Several international studies have indicated that the rates of DM in the Pacific Islander community are disproportionately higher. A recent Samoan study showed increased obesity incidence, which was associated with diet, lifestyle, and genetic factors [14]. This increased obesity incidence can lead to a steady rise in DM. By 2020, a quarter of adults living in Western Samoa were projected to have T2DM [15]. Roughly 25% of the adult population in American Samoa are currently diagnosed with DM, and many more are still undiagnosed [16]. These individuals have a high risk of developing foot complications. In the Pacific Island of Nauru, foot ulcer and amputation rates were approximately 5%-10%, higher than those of Vanuatu and the Solomon Islands [17]. These individuals had poorer diabetic control. Tonga, which is close to Samoa, also has high rates of foot pathologies secondary to poorly controlled T2DM [18]. Early diagnosis and control of blood glucose levels by maintaining a target HbA1c can help manage DM and control DM complications, including DFUs. However, no studies have determined the predictive role of HbA1c in DFU development in the Pacific. Additionally, no studies on DFUs have been conducted in Western Samoa. This limitation broadens the discussion as to whether increased HbA1c levels are associated with DFU development.

This study aimed to identify a specific cutoff point for HbA1c that physicians should be aware of to minimize DFU occurrence. This research hypothesized that high HbA1c levels are strongly associated with DFU development among patients at Tupua Tamasese Meaole (TTM) Hospital in Samoa. This study hopes to lead to better identification of individuals that might potentially develop DFUs and of measures for improving their HbA1c levels.

## Materials and methods

This retrospective unmatched case-control study was conducted at TTM hospital in Samoa between February and May of 2020. Data were collected from the inpatient registry at the internal medicine (IM) ward, as well as the individual clinical files at the records office. The inclusion criteria were both male and female Samoan patients, aged 45-75 years, diagnosed with T2DM for 10 years or longer, and currently on one or more DM medications. Patients from all other ethnicities, newly diagnosed with DM, or not currently on any medication were excluded.

**Figure 1:**
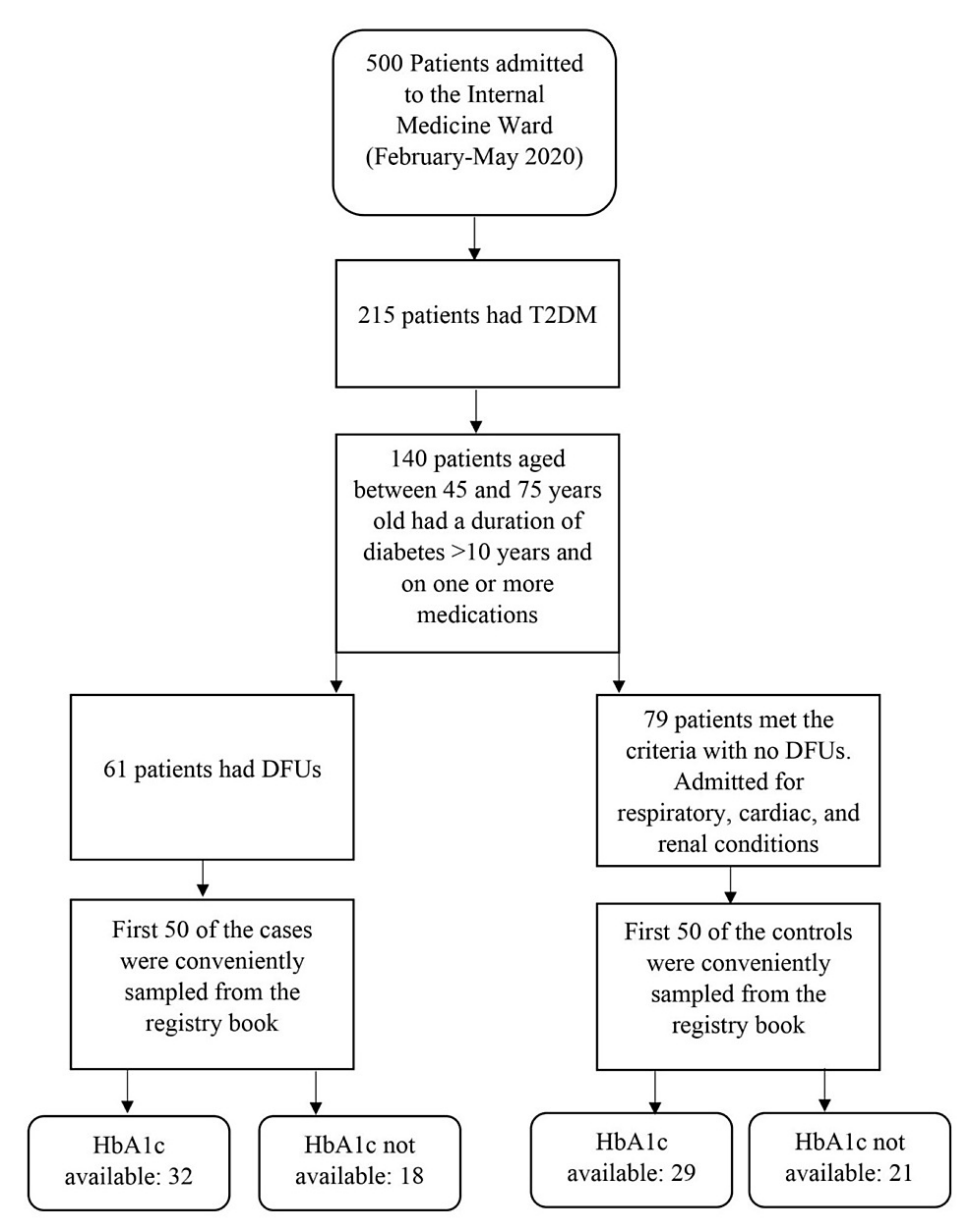
Participant enrollment and sampling technique of the current study. T2DM: type 2 diabetes mellitus; DFUs: diabetic foot ulcers; HbA1c: glycated hemoglobin

Within the study period, 215 out of 590 admitted patients had T2DM. A total of 140 patients were eligible for the analysis. Cases were identified as individuals with DFUs, which were defined in this study as a full-thickness wound through the dermis on the plantar aspect of the foot, right below the ankle [5]. DFUs were found in 61 patients. Among them, 50 were conveniently sampled as cases. The first 50 patients with DFUs were sampled from the inpatient registry book, and their clinical files were obtained from the records office. Meanwhile, controls were selected from the same sample population as the cases, using the same inclusion and exclusion criteria. Contrary to cases, controls did not have DFUs and were admitted for respiratory, cardiac, and renal complications. They accounted for 79 patients. Among them, 50 were conveniently sampled. Likewise, the first 50 patients without DFUs were sampled from the inpatient registry book, and their clinical files were obtained from the records office. However, only 32 cases and 29 controls had HbA1c results in their individual clinical files (Figure 1).

Demographic data, such as sex and age, were collected from the clinical files of each participant. Other collected data were the RBG and HbA1c levels during admission, microvascular disease (retinopathy, neuropathy, and nephropathy), macrovascular disease (ischemic heart disease, stroke, or intermittent claudication), comorbidities (hypertension and hyperlipidemia), ulceration history, and smoking history. This study was approved by the institutional research board of the Oceania University of Medicine and the National Health Service of Samoa.

Descriptive analyses were performed; percentages, medians and interquartile ranges (IQR), and means and standard deviations (SD) were calculated. The normality of data distribution for continuous variables, such as age, RBG, and HbA1c, was determined using the D'Agostino-Pearson normality test. The data were found to be not normally distributed; hence, the Mann-Whitney U test (nonparametric) was used to examine the significant differences of age, RBG level, and HbA1c level between cases (with DFUs) and controls (without DFUs). Furthermore, the receiver operating characteristic (ROC) curve was used to determine the sensitivity and specificity of HbA1c as a test that readily predicts ulceration in patients with T2DM. The area under the ROC curve (AUC) of 50% (0.5) indicates that the test cannot distinguish patients with DFUs from those without DFUs. A value of 70% (0.7) or higher indicates that the test has some utility in distinguishing between these two patient groups. A *P*-value of less than 0.05 was considered statistically significant. Analyses and verification were performed using Microsoft Excel, GraphPad Prism version 8.4.3 (GraphPad Software, San Diego, California, USA), and SPSS version 27 (IBM Corp., Armonk, New York, USA).

## Results

Table 1 summarizes the patient demographics and DM-related characteristics of cases and controls. Males (58%) were more than females (42%) in the case group, whereas females (54%) were more than males (46%) in the control group. The total median age was 58.5 years for the cases and 59 years for the controls. Of note, the control group had more smokers (40%) than the case group (18%). All cases had comorbidities (100%), such as hypertension and hyperlipidemia, as compared with the controls (84%). Furthermore, the cases had more microvascular complications (62%) than the controls (28%). However, the controls had higher macrovascular complications (50%) than the cases (18%). DFU history was more common in the case group (52%) than in the control group (10%). The mean RBG was slightly higher in the case group (18.05) than in the control group (17.43), but the mean HbA1c level was very similar between the two groups (10.6% and 10.7%, respectively).

**Table 1:** Comparison of demographic and T2DM-related characteristics between cases (T2DM with DFUs) and controls (T2DM without DFUs) at at Tupua Tamasese Meaole Hospital. Mean HbA1c calculated from the available HbA1c data of 32 cases and 29 controls. DFU: diabetic foot ulcer; HbA1c: hemoglobin A1c; IQR: interquartile range; RBG: random blood glucose; T2DM: type 2 diabetes mellitus

	T2DM with ulcers (case) n = 50	T2DM without ulcers (control) n = 50
Sex, male (%)	29 (58%)	23 (46%)
Sex, female (%)	21 (42%)	27 (54%)
Age, median (IQR)	58.50 (46–75)	59 (45–75)
Age for males, median (IQR)	59 (49–68)	60 (45–75)
Age for females, median (IQR)	58 (46–75)	59 (47–69)
Smokers, number (%)	9 (18%)	20 (40%)
Frequency of comorbidities, number (%)	50 (100%)	42 (84%)
Frequency of microvascular complications, number (%)	31 (62%)	14 (28%)
Frequency of macrovascular complications, number (%)	9 (18%)	25 (50%)
Frequency of past history of DFUs, number (%)	26 (52%)	5 (10%)
RBG, mean (SD)	18.05 (7.32)	17.43 (8.278)
HbA1c, mean (SD)	10.60 (2.62)	10.70 (2.454)

Table 2 presents the relationship between mean age, RBG levels, and HbA1c levels, which all showed no statistically significant association between the case and control groups (*P* = 0.8703, 0.5331, and 0.9001, respectively).

**Table 2:** Relationship between mean age, RBG, and HbA1c between cases (T2DM with DFUs) and controls (T2DM without DFUs). Mann–Whitney U test, *P* < 0.05 is considered significant. DFU: diabetic foot ulcer; HbA1c: hemoglobin A1c; RBG: random blood glucose; T2DM: type 2 diabetic mellitus

Variable	Study Group	Number	Mean	SD	*P*-value
Age (years)	DFU	50	58.78	6.283	0.8703
	No DFU	50	59.18	7.250	
RBG (mmol/L)	DFU	50	18.05	7.32	0.5331
	No DFU	50	17.43	8.278	
HbA1c (%)	DFU	32	10.60	2.621	0.9001
	No DFU	29	10.70	2.454	

**Figure 2:**
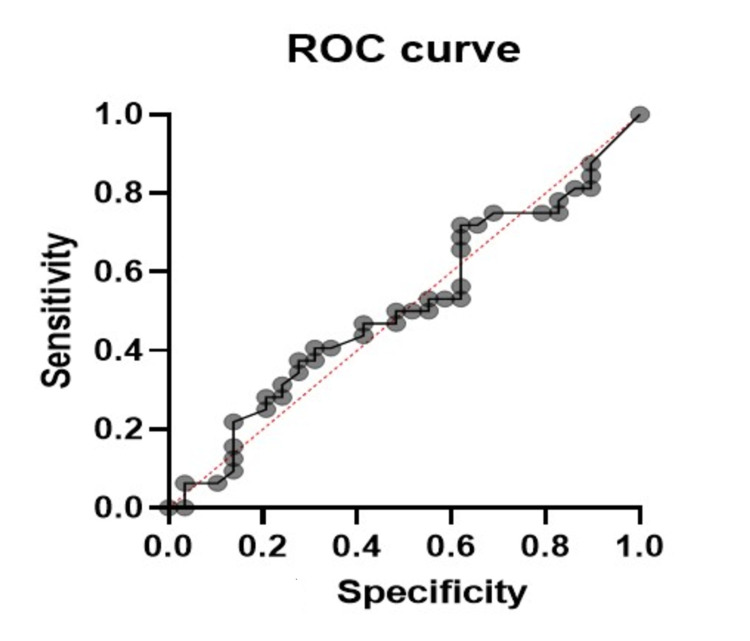
ROC curve showing AUC (51%; 95% CI, 36%–66%; P = 0.8966). ROC: receiver operating characteristic; AUC: area under the ROC curve

Unfortunately, no cutoff point could be established using ROC curve analysis for predicting DFUs because the AUC was only 51% (95% CI, 36%-66%; standard error, 0.075; *P* = 0.8966) (Figure 2).

## Discussion

The results from this sample of patients admitted to the IM ward of TTM hospital in Samoa did not support the expected hypothesis. According to the AUC, the HbA1c test used for predicting DFUs had a roughly equal chance of obtaining true-positive and false-positive test results, and no cutoff point could be established. Therefore, the HbA1c level is not singly predictive of DFUs in these patients. This outcome is not consistent with previous studies in Sudan, Australia, and the USA, which showed that increasing HbA1c levels are associated with an increased risk of foot ulceration [19-21,13]. An extensive literature review revealed that no studies have examined the role of HbA1c in predicting foot ulcer development in the Pacific Islands. The results of the present study may be explained by the convenience sampling scheme and the small sample size, which may have decreased the power to detect statistically significant differences. Studies employing a random sampling technique and a larger sample size are recommended in the future.

In the present study, age distribution was not significantly associated with DFUs. The average age for both groups was very similar (case, 58.78; control, 59.18), indicating no differences between the two groups. This result is not consistent with numerous studies in the literature, where age is a significant risk factor of DFU development [22]. A recent case-control study in Yemen reported that patients aged above 55 years had the highest tendency to have DFUs, followed by those aged 36-55 and 26-35 years [23]. Similarly, a study from Bangladesh reported that people aged below 50 years were protective against DFU development, whereas those aged above 50 years were associated with DFU development [24]. The findings of the present study may have resulted from the strict inclusion criteria of patients aged between 45 and 75 years and having a history of DM for 10 years or longer. The critical point may not be the patients’ age but rather how long they have had DM. The longer the duration of their DM, the greater the chances of having DM complications. Two Australian studies emphasized that the duration of DM is an important predictor of DM-related foot pathology [25,26]. Prospective studies in Samoa could essentially broaden the age range or limit diabetic history to less than 5 years to assess for any observable relationship between age and ulcer development.

In addition, the RBG level was not significantly associated with DFUs. The RBG level was obtained during admission for both groups. Frequently, patients with diabetes that require hospitalization are already quite ill, and a derangement in their blood glucose levels is expected, especially if patients are septic. These patients generally have elevated blood glucose levels [27]. To our knowledge, no studies within Australia, New Zealand, or the Pacific Islands have examined the relationship between RBG and DFUs.

Interestingly, patients with a history of DFUs were more common in the case group (52%) than in the control group (10%). This result is consistent with numerous Australian studies, which observed that individuals who presented with a DFU in a healthcare service were more likely to have had a previous DFU experience [28,29]. Patients with a previous DFU are likely to have peripheral neuropathy and a foot deformity. Having an insensate foot predisposes them to microtrauma, skin breakdown, and ultimately, DFU. A 10 g monofilament has been shown to detect diabetic peripheral neuropathy and predict DFUs [13]. This could be used in conjunction with glycemic monitoring to detect DFUs in a setting such as Samoa.

This study has several strengths. First, this study is the first in the Pacific Islands to assess the predictive role of HbA1c in DFU development. Second, it has highlighted the overall trend of poorer T2DM control in Samoa. The RBG and HbA1c levels were markedly elevated in both groups. Considering that cases and controls were both sampled from TTM hospital in Samoa, better glycemic control may reduce the number of hospitalizations. It also points to the state of primary care measures to manage patients with T2DM in the community. Managing newly diagnosed patients appropriately will prevent future complications, such as DFUs.

In contrast, this study also has several limitations that need to be considered when understanding the results. First, the sample size is relatively small, similar to earlier studies. The study in Sudan used a relatively small sample size [19]. Conversely, the studies in Australia and the USA used larger samples; however, they used a longitudinal study design [20,21]. Second, convenience sampling was used to collect cases and controls from the inpatient registry book, implying a selection bias. This sampling scheme was chosen because of the ease of clinical file retrieval. Prospective studies are recommended to implement a randomized sampling scheme. Third, the HbA1c results were missing in several individual clinical files for both cases and controls because of the lack of reagents at the TTM hospital laboratory during specific weeks, owing to the coronavirus disease 2019 (COVID-19) restrictions in Samoa. The patients without HbA1c values could not be excluded initially because these values were unavailable in the inpatient registry of the IM ward. The individual clinical files were searched at the records office, and the HbA1c values were then found on the laboratory results page. This task is time-consuming, highlighting the potential struggle of obtaining laboratory results in a resource-limited setting such as Samoa. Last, the interpretation of the results was entirely dependent on the interpretation of written clinical notes. Thus, the clinical staff was consulted where it was possible to corroborate findings in the written clinical notes.

## Conclusions

This study is the first in the Pacific Islands to evaluate whether HbA1c could be used as a marker to predict DFU development. High HbA1c levels were not associated with DFU development in the sample obtained from an IM ward in Samoa. No optimal cutoff point for HbA1c could be determined. Hence, HbA1c may not be singly predictive of DFUs. However, an elevated HbA1c level was noted in both groups owing to poor long-term glycemic control. Improving glycemic control is recommended in this patient group. Well-established methods for predicting DFUs such as cutaneous sensation via 10 g monofilament should be considered in a resource-limited setting such as Samoa. Further exploration of these findings is recommended by improving upon the existing study design. The HbA1c test may be considered in addition to the 10 g monofilament as a multifactorial measure for improved prediction of DFUs in future studies.
